# Complete Genome Sequence of a Field Isolate of Classical Swine Fever Virus Belonging to Subgenotype 2.2 from India

**DOI:** 10.1128/genomeA.00288-18

**Published:** 2018-06-14

**Authors:** Neelam Tomar, Veena Sharma, Jeny K. John, Menaka Sethi, Pradeep K. Ray, Rahul S. Arya, Tareni Das, G. Saikumar

**Affiliations:** a Division of Pathology, Indian Veterinary Research Institute, Izatnagar, Uttar Pradesh, India; b Department of Bioscience & Biotechnology, Banasthali University, Vanasthali, Rajasthan, India; c Veterinary Pathology, ICAR Research Complex for Eastern Region, Patna, Bihar, India; d Department of Veterinary Pathology, College of Veterinary Science, CAU, Aizawl, Mizoram, India

## Abstract

The complete genome sequence of classical swine fever virus (CSFV) strain CSFV-UP-BR-KHG-06, from genotype 2.2, was determined. Comparative analysis based on the amino acid sequence of some important B-cell epitopes, T-cell epitopes, glycosylation sites, and conformational residues showed the striking differences between the group 2 virus KHG-06 and the vaccine strains HCLV/India and C-strain.

## GENOME ANNOUNCEMENT

Classical swine fever (CSF), which causes heavy losses in the swine industry, is one of the Office International des Epizooties (OIE) notifiable diseases caused by classical swine fever virus (CSFV). CSFV is a positive-sense RNA virus belonging to the genus Pestivirus within the family *Flaviviridae*. The CSFV genome is approximately 12.3 kb in length and encodes a single polyprotein that is cotranslationally and posttranslationally cleaved to form mature structural and nonstructural proteins (1). Based on the nucleotide sequences of partial 5ʹ nontranslated region (NTR) and NS5B and partial or whole E2, CSFV isolates have been divided into three genotypes and 11 subgenotypes (1.1 to 1.4, 2.1 to 2.3, and 3.1 to 3.4) (2–4). In India, subgenotype 2.2 is predominantly circulating, but information regarding the full-genome sequence of genotype 2.2 viruses from India is very limited. In spite of vaccination, several CSFV outbreaks have occurred. In order to access the more detailed knowledge about the immune escape of CSFVs, it was decided to study the full-length genome of dominantly circulating 2.2 genotype viruses in India.

Total RNA was extracted from a pooled tissue homogenate of tonsils, lymph node, and kidney using the TRIzol reagent (Invitrogen), according to the manufacturer’s instructions. The full nucleotide sequences were obtained from 11 overlapping fragments amplified by reverse transcription-PCR and were assembled and manually edited to produce the final genome sequence. The complete genome sequence of isolate CSFV-UP-BR-KHG-06 (KHG-06) is composed of 12,298 nucleotides (nt), with a 5ʹ untranslated region (UTR) of 373 nt and a 3ʹ UTR of 227 nt. A single large open reading frame (ORF) is 11,697 nt between nucleotide positions 374 and 12070 and is capable of coding for a polyprotein of 3,898 amino acids.

The BLAST result of the nucleotide sequence of isolate KHG-06 showed similarity with isolates NVD-11 (98%), SKN-11 (98%), LAL-290 (97%), Strain-39 (93%), Bergen (92%), and, on the basis of amino acid sequence similarity, with NVD-11 (98%), SKN-11 (98%), Strain-39 (95%), GD53/2011 (95%), and Bergen (92%). A phylogenetic tree was constructed using the MEGA 6.01 program (5); on the basis of the full-genome sequences of CSFV strains deposited in GenBank, it was revealed that isolate KHG-06 belongs to subgenotype 2.2.

Comparative analysis based on amino acid sequences of some important B-cell epitopes, T-cell epitopes, glycosylation sites, and conformational residues showed the striking differences between the group 2 virus KHG-06 and the vaccine strains HCLV/India and C-strain. It also revealed a close antigenic relationship with Chinese strains, especially recombinant isolate Strain-39. In the Erns protein of all analyzed isolates, a unique *N*-linked glycosylation site is identified at position 367 (NLTE) along with other genogroup 2.2 isolates, but it is absent in most of the group 1 isolates, including vaccine strains, C-strain, and HCLV/India. The new genome sequences may contribute to further understanding of the phylogeny and antigenic variation in CSFV.

### Accession number(s).

The complete genome sequence of CSFV isolate CSFV-UP-BR-KHG-06 has been deposited in GenBank under the accession number KC533775.
